# Hollow fiber bioreactor allows sustained production of immortalized mesenchymal stromal cell-derived extracellular vesicles

**DOI:** 10.20517/evcna.2023.76

**Published:** 2024-05-14

**Authors:** Sergio G Garcia, Marta Sanroque-Muñoz, Marta Clos-Sansalvador, Miriam Font-Morón, Marta Monguió-Tortajada, Francesc E. Borràs, Marcella Franquesa

**Affiliations:** ^1^REMAR-IGTP Group, Health Science research Institute Germans Trias i Pujol (IGTP), Can Ruti Campus, Badalona 08916, Spain.; ^2^Department of Cell Biology, Physiology and Immunology, Universitat Autònoma de Barcelona (UAB), Bellaterra 08193, Spain.; ^3^Department of Biochemistry and Cell Biology, Universitat Autònoma de Barcelona (UAB), Bellaterra 08193, Spain.; ^4^Department of Cell Biology, Physiology and Immunology, Universitat de Barcelona (UB), Barcelona 08028, Spain.; ^#^Authors contributed equally.

**Keywords:** Mesenchymal stromal cells (MSCs), extracellular vesicles (EV), advanced therapeutic medical product (ATMP), Hollow fiber bioreactor (HFB), immortalization, Large-scale, therapy

## Abstract

**Aim:** Mesenchymal stromal cell-derived extracellular vesicles (MSC-EVs) have been reported to hold great potential as cell-free therapies due to their low immunogenicity and minimal toxicity. However, the large doses of MSC-EVs that are required for their clinical application highlight the urgency of finding a large-scale system for MSC-EV manufacture. In this study, we aimed to set up a hollow fiber bioreactor system for the continuous homogenous production of functional and high-quality MSC-EVs.

**Methods:** MSC lines from two donors were immortalized (iMSC) and inoculated into hollow fiber bioreactors. Throughout 4 weeks, conditioned medium was daily harvested. iMSC-EVs were purified and characterized for content, immunophenotype, size, and functionality and compared to 2D cultured iMSC.

**Results:** The iMSC inoculated into the bioreactor remained viable during the whole culture period, and they maintained their MSC phenotype at the end of EV production. Our results showed that the bioreactor system allows to obtain 3D-cultured iMSC-derived EVs (3D-EVs) that are comparable to flask (2D)-cultured iMSC-derived EVs (2D-EVs) in terms of protein and lipid content, size, and phenotype. We also confirm that 3D-derived EVs exhibit comparable functionality to 2D-EVs, showing pro-angiogenic potential in a dose-dependent manner.

**Conclusions:** These findings suggest that setting up a hollow fiber bioreactor system inoculating immortalized MSC lines facilitates the large-scale, functional, and high-quality production of iMSC-EVs. Our results emphasize the great potential of this production methodology to standardize EV production in the pursuit of clinical applications.

## INTRODUCTION

Mesenchymal stromal cells (MSCs) hold potential as a cell therapeutic strategy in many diseases. MSCs exert immunomodulatory and regenerative therapeutic effects, which, combined with their relative ease of culture and stable phenotype in vitro, explain their wide use for cell therapeutic approaches involving alloimmunity, autoimmunity, and inflammation^[1]^. Notably, MSCs have been employed in various clinical trials to address diverse pathologies, including graft-versus-host disease (GVHD)^[2]^, Crohn’s disease^[3]^, and multiple sclerosis^[4]^. Nevertheless, there are drawbacks associated with using whole cells, including issues with cell culture scalability and cryopreservation, as well as other safety concerns such as embolism or the potential risk of uncontrolled proliferation^[5,6]^.

In this scenario, several studies have highlighted the relevance of MSC-derived extracellular vesicles (MSC-EVs) and their advantages over using whole cells for their clinical application^[7,8]^. These nanosized membranous structures are effective carriers for intercellular communication and cargo transportation, thus enhancing their therapeutic potential^[9]^. MSC-EVs offer attractive traits for cell-free therapies, such as low immunogenicity, minimal toxicity, and responsiveness to post-administration microenvironment changes^[10]^. Moreover, EVs are non-living and non-replicative and have a transient presence in the body^[11]^.

However, a concern in the generation of replicable doses of MSC therapy (and therefore in MSC-EVs therapy) is cell senescence after successive cell culture passages^[12]^, giving rise to decreased immunosuppressive properties and lower replicative capacity^[13-15]^. Immortalization of primary MSC may overcome senescence and allow the generation of genetically stable immortalized MSC lines (iMSCs) that could also circumvent MSC culture heterogeneity. Transfection and expression of the human telomerase reverse transcriptase (hTERT) gene was described as a method to induce MSC cell lines characterized by an enhanced cell proliferation rate, reduced senescence, and maintained functional potential, that could potentially lower batch-to-batch functional variability^[16,17]^. Further studies described the application of viral proto-oncogenes such as SV40 and human papilloma virus (HPV) E6/E7 genes in combination with hTERT^[18]^ with high success in inducing proliferation and avoiding senescence, while contesting the potential of hTERT to immortalize human adipose-derived stromal cells. However, the use of viral transcripts raises safety concerns over their clinical application and reduces their translational potential in comparison with hTERT immortalized MSC.

Complementing immortalization, the development of large-scale systems allowing continuous production of functional and high-quality EVs may solve limitations associated with classical culture methods, providing enhanced scalability, reduced manual handling, and easy monitoring and control of culture parameters^[19]^. In recent years, significant advancements have been made in three-dimensional culture techniques to overcome existing limitations. Zhang *et al.* seeded human bone marrow-derived MSC within 3D collagen scaffolds to enhance EV production, demonstrating an improvement in repair function in rats after brain injury compared to classical 2D-derived EVs^[20]^. On the other hand, Haraszti *et al.* developed a scalable microcarrier-based 3D culture to double the EV yield, counting with a large total surface area and being capable of culturing millions of MSC^[21]^. However, these three-dimensional approaches faced challenges regarding the continuous collection of culture media and were limited to single or few isolations of conditioned media. Despite the increased rate of EV production, concerns persisted regarding plastic waste and economic and time costs. In this regard, hollow fiber bioreactors emerge as a promising solution for large-scale EV manufacturing. These systems provide a three-dimensional (3D) living environment that more closely mimics *in vivo* cell spatial distribution features and interactions^[22]^. Specifically, cells are grown inside a small cartridge containing thousands of hollow fibers where cells get attached. Fibers are manufactured with pores that allow the flowthrough of small molecules and nutrients toward the cellular compartment, and enable the culture of cells in a small volume of supernatant. This fiber setting increases cell growth surface area, allowing high-density cell cultures in a reduced space and volume, easing downstream isolation of EVs from the highly concentrated conditioned medium^[23]^. Using this system, cell culture and supernatant harvesting are streamlined, reducing economic and time costs, while limiting downstream processing efforts by harvesting highly concentrated EV-enriched supernatants, easing large-scale EV manufacturing.

Considering the potential of implementing large-scale systems to produce EVs for therapeutic approaches and the potential advantages of using iMSC as an EV source, in this study, we aim to establish iMSC culture in a hollow fiber bioreactor to produce iMSC-EVs and comprehensively characterize them. For this purpose, we will generate and characterize hTERT immortalized MSC lines and thoroughly study secreted EVs in 2D and 3D systems, including their quantification, characterization and function between systems and longitudinally in the hollow fiber bioreactor.

## METHODS

### Sample collection and Ethics statement

Mesenchymal stromal cells (MSCs) were obtained from human umbilical cord Warthon’s Jelly from healthy donors from Hospital Universitari Germans Trias i Pujol of Badalona (Spain) following standard operation procedures^[24]^. Informed consent was obtained from all subjects and the study protocols were approved by the Clinical Research Ethics Committee of our institution (CEIC: EO-10-016 and EO-12-022) and conformed to the principles outlined in the Declaration of Helsinki (BMJ 1991; 302:1994). All samples were anonymously analyzed.

### Cell culture and immortalization procedures

Wharton’s Jelly MSCs were seeded in 175 cm^2^ culture flasks at 2,000 cells/cm^2^, in 15 mL of minimum essential medium-α (Lonza, Verviers, Belgium) supplemented with 1% penicillin (100 IU/mL, Cepa S.L., Madrid, Spain) and streptomycin (100 mg/mL, Normon Laboratories S.A., Madrid, Spain), 1% L-glutamine (Sigma Aldrich, St. Louis, MO, USA), and 10% heat-inactivated fetal bovine serum (Lonza); at 37 ºC, 5% CO_2_, and 95% humidity.

To generate the cell lines, primary MSCs from two donors with expression of classical MSC surface markers and proven immunosuppression capabilities, as extensively detailed in a previous publication^[24]^, were transfected with the human telomerase reverse transcriptase (hTERT) following a previously published protocol^[25]^. Primary cells were transfected between passages 8 and 10. The retroviral vector used was pBABE-hygromycin-human telomerase reverse transcriptase (Plasmid#1773, Addgene). All the experiments were performed with both iMSC lines maintained under the same culture conditions as primary MSCs.

Primary lung-derived fibroblasts were seeded in 75 cm^2^ culture flasks at 2,000 cells/cm^2^, in 10 mL of Dulbecco’s Modified Eagle Medium (Thermoscientific) supplemented media with 1% penicillin (100 IU/mL, Cepa S.L., Madrid, Spain) and streptomycin (100 mg/mL, Normon Laboratories S.A., Madrid, Spain), 1% L-glutamine (Sigma Aldrich, St. Louis, MO, USA), and 10% heat-inactivated fetal bovine serum (Lonza), at 37 ºC, 5% CO_2_, and 95% humidity.

### Characterization of iMSCs

#### iMSC/MSC proliferation and hTERT expression

To confirm constant exponential growth of the iMSCs, the cells were kept in culture and followed for approximately 200 days. Weekly cell counting was performed to establish the number of doublings in the passage and the cumulative doublings calculated as 
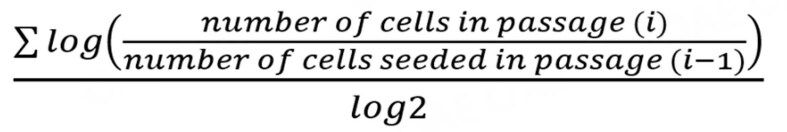


Non-immortalized MSCs growth rate was followed until the cells reached the stationary phase.

To determine hTERT expression in cells (primary MSC and iMSC) and in iMSC-derived EVs, RT-PCR and qPCR were performed. To analyze hTERT transference to primary cell cultures, lung-derived primary fibroblasts were cultured in the presence of iMSC-EVs. Briefly, 50,000 fibroblasts were seeded in 24 well plates (Cultek, #153524) and left overnight to ensure cell attachment in a cell culture incubator at 37 ºC, 5% CO_2_, and 95% humidity. Then, media was changed to supplemented DMEM with iMSC-EVs corresponding to 1,000,000 producing cells and incubated for 6 h at 37 ºC. After incubation, fibroblast cultures were washed, and total RNA was extracted.

In all samples, total RNA was extracted using RNeasy mini Kit (QIAGEN) and DNase I treatment (RNase-free DNase Set QIAGEN) was performed. RNA was eluted in RNase-free water and quantified using NanoDrop^TM^ 1000 spectrophotometer (Thermo Fisher Scientific). Whole RNA (100 ng) was reverse-transcribed using random hexamers and the iTaq Probes One-step Kit (Biorad) according to the manufacturer´s protocol. qPCR was performed in triplicates using 1.5 ng of RNA for all samples, hTERT-specific primers (Forward: 5’-CACTCCTGGGGTCACTCAG-3’. Reverse: 5’-CTTGAAGTCTGAGGGCAGTG-3’), and the PowerUp SYBR Green Master Mix (Thermo Fisher Scientific) in the LightCycer 480 II system (Roche) and analyzed using the LightCycler®480 software version 1.5.0 (Roche). The crossing point (CP) of the amplification curves was calculated, and the expression of hTERT was compared using the 2^-ΔCt^ method. CP values > 35 were considered uncertain or under the limit of detection.

#### Cytogenetic and immunophenotype characterization

Cytogenetic analysis was performed by conventional G-banding karyotypes using standard procedures. Colcemid was added to the iMSC cultures to arrest cells in metaphase. After centrifuging, hypotonic solution (KCl, 0.075M) was added and washes were performed with Carnoy’s solution to break the cytoplasmic membranes. Chromosomes were fixed in the spindle to obtain the cells that were in interphase and metaphases. The G band pattern was achieved with Wright dye. Karyotypes were analyzed and described following the International System for Human Cytogenetic Nomenclature (ISCN 2020)^[26]^ by the Cytogenetics Unit of Catalan Institute of Oncology.

iMSC were phenotypically characterized by studying canonical MSCs surface markers. Cells were labeled with anti-CD90-PE-Cy7 (BD, clone 5E10), -CD73-PE (BD, clone AD2), -CD166-PE (BD, clone 3A6), -HLA-ABC-FITC (BD, clone G46-2.6), -CD34-PE (BD, clone 563), -CD14-FITC (BD, clone M5E2), -HLA-DR-FITC (BD, clone G46-6), -CD19-V450 (BD, clone 4G7), -CD46-FITC (BD, clone 2D1), -CD49c-PE (BD, clone C3 II.1), -CD49e-FITC (BD, clone IIA1), -CD24-APC (Invitrogen, clone SN3 A5-2H10), -CD10-PE (BD, clone HI10a), -CD106-FITC (BD, clone 51-10C9), -CD29-PE (BD, clone HUTS-21), -VEGFR2-PE (RD Systems, clone 89106), -CD49d-FITC (Immuno Tools, clone PS/2), -CD59-APC (Biolegend, clone H19), -CD117-PE (BD, clone YB5.B8), -CD184(CXCR4)-PE (BD, clone 12G5) for 20 min at room temperature (RT), washed with 2 mL of FACSFlow and centrifuged at 400 × *g* for 5 min before acquisition. Data were acquired in a Canto II flow cytometer (BD Biosciences) and analyzed using FlowJo_v10.7 software (TreeStar, Ashland, OR).

#### Proliferation assay

T cell proliferation assays were performed as previously described^[24]^. Briefly, blood samples were obtained after informed consent from healthy volunteers in EDTA tubes. After Ficoll Hypaque Plus^TM^ (GE Healthcare, Uppsala, Sweden) density gradient centrifugation, T cells were isolated by negative selection using the EasySep^TM^ Human T cell Enrichment Kit (StemCell Technologies, Grenoble, France), labeled with Carboxyfluorescein succinimidyl ester (CFSE; Molecular Probes, Leiden, The Netherlands) and stimulated with anti-CD2, -CD3 and -CD28 coated microbeads (bead 1:10 T cell ratio: Pan T Cell Activation Kit; Miltenyi Biotech). Labeled T cells without stimulation were used as a negative control of proliferation. To test MSC and iMSC T cell proliferation inhibition capacity, different concentrations (1,250, 5,000 and 20,000 cells/well) of both types of MSC were seeded into flat-bottomed 96-well plates, and after attachment, 50,000 T cells were added. Wells without MSC were used to establish T cell basal proliferation. Finally, T cell proliferation was measured after 3.5 days in an LSR Fortessa Analyzer (BD Biosciences) and analyzed using FlowJo_v10.7 software (TreeStar, Ashland, OR).

### Hollow fiber bioreactor setting-up and cell inoculation

#### Hollow fiber bioreactor system

**Figure 1:**
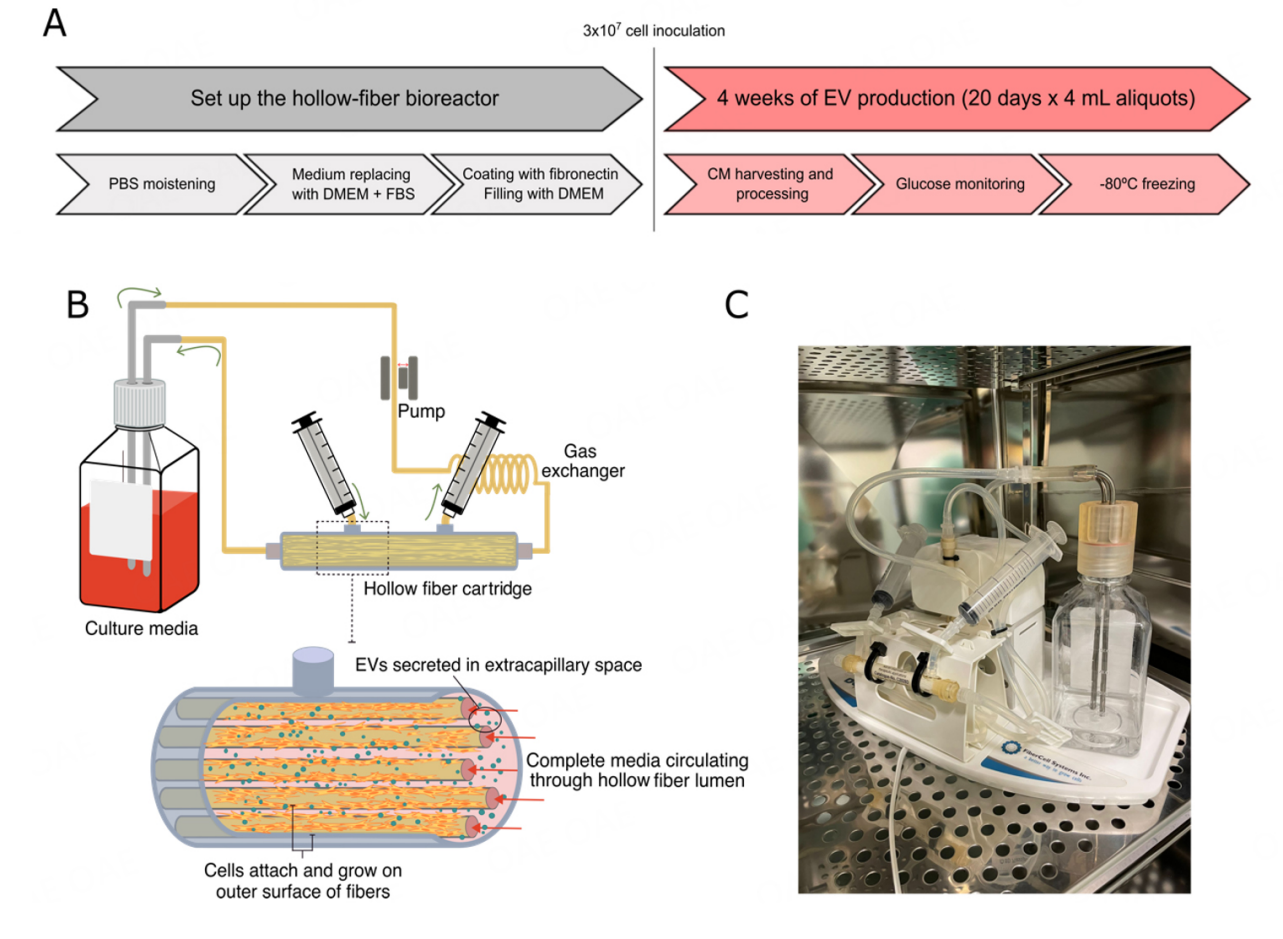
Hollow fiber bioreactor system. (A) Graphical representation of bioreactor-based workflow for iMSC-EVs production. Prior to seeding the cells, the cartridge was moistened with PBS and coated with fibronectin. After that, in the reservoir bottle, PBS was replaced with culture media. Once seeded, a daily 4 mL aliquot was obtained, processed, and frozen for future analysis as described in the text; (B) Schematic representation of a hollow fiber bioreactor-based 3D culture system. It consists of a cartridge with hollow fibers, through which the circulating medium will pass, and an extracapillary space where the cells will attach and release the EVs. The culture medium circulates through a circuit, pushed by a pump; (C) Photograph of our hollow fiber bioreactor.

The hollow fiber bioreactor was set up and operated according to the manufacturer’s instructions^[27]^, as shown in Figure 1. Briefly, on day 1, the circuit was perfused with PBS to run through the system for 24 h at 37 ºC, at a flow rate of 10 (45 mL/min), and 15 mL of PBS was injected into the extracapillary space (ECS) to fill it. On day 2, the ECS was coated with a solution of 1 mg of fibronectin (Human Plasma Fibronectin Purified Protein FC010-5MG, Millipore) in 20 mL PBS to enhance cell attachment. Fibronectin solution was injected using one of the ECS syringes and spread throughout the cartridge fibers by mobilizing the solution from one syringe to the other 6 times. Excess volume was removed by running the solution through the pores to the circulating PBS. After removing the fibronectin buffer, the ECS was filled with 4 mL of chemically defined medium (MSC Brew, Miltenyi Biotec) and the circulating PBS was replaced with 125 mL of high glucose Dulbecco's Modified Eagle Medium (Thermoscientific) supplemented with 1% penicillin (100 IU/mL, Cepa S.L., Madrid, Spain) and streptomycin (100 mg/mL, Normon Laboratories S.A., Madrid, Spain), and 1% L-glutamine (Sigma Aldrich, St. Louis, MO, USA). This medium was kept through the system for 24 h at a flow rate of 10 and was finally replaced with 125 mL of fresh supplemented high glucose DMEM, as previously defined, with 10% heat-inactivated fetal bovine serum (Lonza). Taking advantage of the filtration capacity of the hollow-fiber system, cells were cultured in the cartridge/extracapillary space (ECS) in chemically defined media, while the circulating space was filled with serum-supplemented media to feed the cells. A 20 kDa molecular weight cut of porous size was chosen for the cartridge fibers to guarantee EV retention in the cartridge and avoid the flowthrough of serum-derived extracellular particles to the isolated supernatants.

To perform the experiments, two bioreactors were set up. Cells were expanded in 175 cm^2^ culture flasks, as explained in the “Cell culture and immortalization procedures” section, until 90% of cell confluency was reached. At that time, cells were harvested and resuspended in a final volume of 20 mL of chemically defined medium. Finally, cells were transferred to the hollow fiber bioreactor (FiberCell Systems) at 3 × 10^7^ cells/cartridge (C2025D, 20-kD MWCO, 450 cm^2^, polysulfone fiber cartridge; FiberCell Systems) following the manufacturer’s procedure^[27]^.

#### Hollow fiber bioreactor cell culture and monitoring

For cell culture in the hollow fiber bioreactor, the media volume in the reservoir bottle was 500 mL and circulated at a flow rate of 10 (45 mL/min). A 300 μL aliquot of medium from the reservoir and ECS was collected daily to monitor glucose, lactate dehydrogenase content, and metabolites. Glucose levels were quantified using a GlucCell^TM^ Glucose Monitoring System (CESCO Bioengineering), and lactate dehydrogenase was assessed with CyQUANT™ LDH Cytotoxicity Assay (ThermoScientific) commercial kit. Additional screening of metabolites in the supernatant was carried out by nuclear magnetic resonance (NMR). In short, 250 μL of cell culture supernatant from iMSC grown in the hollow fiber bioreactor was shipped in dry ice to Biosfer Teslab (Reus, Spain) for metabolite analysis using nuclear magnetic resonance. Week 1 and week 4 supernatant and circulating medium of iMSC cultured in the hollow fiber bioreactor was analyzed using chemically defined and DMEM high glucose basal medium as controls.

250 mL of medium from the reservoir was renewed each time whenever glucose levels in ECS decreased by 20% of the initial value, and 4 mL of fresh chemically defined medium were daily injected into the ECS to obtain 4 mL of EV-rich cell conditioned medium (CM) for subsequent EV isolation. Each CM aliquot was centrifuged 400 × *g* for 5 min and 2,000 × *g* for 10 min. All centrifuged CM were stored at -80 ºC for future EV isolation and the cell pellet from the first centrifugation step was used to count detached alive and dead cells by staining with methylene blue and counting in a hemocytometer.

On the last day of EV production, PBS was injected into the ECS instead of fresh chemically defined medium, and cells were trypsinized twice with 6.5 mL of trypsin-EDTA 0.25% (Lonza) for 10 min at 37 ºC. The detached cells were pushed with PBS, trypsin was neutralized with complete medium, and the cell suspension was centrifuged at 400 × *g* for 5 min. The recovered cells were phenotypically characterized to see whether iMSC surface marker profile would be altered due to long-term 3D culture.

### Isolation and characterization of iMSC-EVs

#### EV isolation by size exclusion chromatography

Cell expansion and EV isolation from MSC cultured in flasks (2D cultures) were performed according to previous publications^[28]^. To isolate EVs from the bioreactor (3D cultures), each centrifuged CM was thawed and concentrated using 100 kDa Amicon units (Millipore) by centrifugation at 2,000 × *g* for 30 min. Concentrated CM (CCM) was transferred to low binding 1.5 mL tubes (Eppendorf), and EV purification was performed by SEC as previously described^[28]^. SEC fractions 3 to 8 were analyzed by bead-based flow cytometry to identify classical EV marker CD63 and MSC marker CD90 as previously described^[28]^. SEC fractions protein content was assessed by nanodrop. The SEC fractions showing the highest median fluorescence intensity for both markers were pooled and frozen at -80 ºC.

#### Nanoparticle tracking analysis

Average size distribution and particle concentration analyses of the samples were performed by NTA on the ZetaView × 30 platform equipped with the software version 8.05.16 (ParticleMetrix, Meerbusch, Germany). 50 µL of sample was diluted in PBS and 1 mL of diluted sample was loaded into the flow cell and measured. 520 nm laser was used for the measurements of the EVs. Particle sizes and numbers of all 11 positions were recorded and calculated as the mean of the results. The following settings were used: Positions: 11; cycles: 5; quality: medium; min brightness: 30; min area: 10; max size: 1,000; trace length: 15; nm/class:5; sensitivity: 80; shutter: 100; and framerate: 30.

#### Total protein and calnexin quantification in EV fractions

Total protein content of each EV pool was assessed by micro-bicinchoninic acid assay (µBCA) (Micro BCA™ Protein Assay Kit, ThermoScientific) following the manufacturer’s instructions.

To discard the presence of contaminant non-EV markers (e.g., calnexin) and confirm the presence of CD63 in all EV samples, dot blot assays were performed. 2 µL of the samples were placed on a nitrocellulose membrane and non-specific sites were blocked with BSA/ TBS-T (TBST, 0.1% BSA) (Sigma) for 1 h. Then, the sample was incubated for 30 min with primary antibody (1:250 dilution for calnexin, 1:300 dilution for CD63) (Santa Cruz Biotech,) and washed (3 times, 5 min) with TBS-T (TBS, 0.05% Tween 20). The membrane was incubated with the secondary antibody for 30 min (IRDye 800 CW Goat anti-Mouse IgG Secondary Antibody, LICOR), washed 3 times with TBS-T (1 × 15 min, 2 × 5 min) and once with TBS. Finally, it was read on the Odyssey^®^ DLx imaging system (LI-COR). The whole experiment was carried out at RT.

#### Quantification of lipid concentration in EV fractions

Total lipid content of each EV pool was assessed by sulfo-phospho-vanillin (SPV) lipid assay following an established protocol^[29]^. Briefly, 40 μL of EV sample or liposome standard were mixed with 200 μL of 96% sulfuric acid and incubated for 20 min. After that, 120 μL of phospho-vanillin reagent were added to each condition and 280 μL of each EV-sample/standard was transferred to a 96-well flat-bottom plate and incubated at 37 °C for 1 h. Finally, the 96-well plate absorbance (540 nm) was measured in a luminometer LB 960 plate reader (Berthold Technologies).

#### Multiplex bead-based flow cytometry

Surface antigen expression of each isolated EV pool was further analyzed using the MACSPlex human Exosome Kit (Miltenyi Biotec). In brief, each EV sample (corresponding to 4 μg of protein) was incubated with 15 μL of MACSPlex Exosome Capture beads overnight at RT in rotation. After that, 15 μL of cocktail comprising MACSPlex Exosome Detection Reagent for CD9, CD63, and CD81 was added to each EV sample. Bound EVs were identified by different fluorescence intensities detected in the FITC and PE channels by flow cytometry. Single analyte MFIs were normalized to the mean MFI of tetraspanins CD9, CD63 and CD81 according to the manufacturer's instructions to calculate their expression.

#### Super-resolution microscopy

Super-resolution microscopy was used to determine the differential expression of tetraspanins in 2D and 3D culture-derived EV samples.

Super-resolution microscopy images were captured using the Nanoimager S Mark II microscope from ONI (Oxford Nanoimaging, Oxford, UK). The acquisition of iMSC-EVs samples followed established protocols^[30,31]^. EVs were labeled with the EV profiler kit (ONI, UK). For the staining process, 7 µL of EV sample were incubated overnight with 2 µL of blocking buffer and 1 µL of antibody mix. Images were acquired in dSTORM mode sequentially, using total internal reflection fluorescence (TIRF) mode. The acquired single-molecule data were filtered using NImOS software (v.1.18.3, ONI), and further processed using the drift correction pipeline (version 0.2.3)^[31]^ on the CODI (the Collaborative Discovery) online analysis platform available at https://alto.codi.bio.

#### Cryo-transmission electron microscopy

Pooled EV-enriched fractions were selected for Cryo-transmission electron microscopy (Cryo-TEM) microscopy to analyze the EV size and morphology. Vitrified specimens were prepared and analyzed with a Jeol JEM 2011 transmission electron microscope as previously described^[24]^. Images were recorded on a Gatan Ultrascan 2000 cooled charge-coupled device (CCD) camera with the Digital Micrograph software package (Gatan).

#### Matrigel-based tube-like formation assay

The angiogenic capacity of human umbilical vein endothelial cells (HUVEC) was tested using specifically adapted 96w-plates (μ-plates angiogenesis 96 well, Ibidi, Germany). Wells were pre-coated with 10 µL of Matrigel (Corning, NY, USA), and incubated for 10 min at RT and 20 min at 37 °C. Then, 10,000 HUVEC/well were seeded and cultured in complete EGM-2 (Lonza) without VEGF with iMSC-EVs from 3D cultures from 80,000 or 160,000 producing cells. The cells were incubated for 4 h at 37 °C and images were taken using a LEICADMI6000B microscope (Leica) at 10x and analyzed using the Image J-Angiogenesis analyzer tool (ImageJ, NIH) [Supplementary Figure 1].

### Statistical analysis

Data normality was tested by the Shapiro-Wilk test and data were analyzed for statistical significance by the Mann-Whitney test to compare means between two groups, one-way ANOVA followed by the Kruskal-Wallis test to compare multiple groups, and ordinary two-way ANOVA followed by Bonferroni’s multiple comparison test for multiple comparisons using Graph-Pad Prism software (9.0 version). Data were considered statistically significant when *P* < 0.05.

## RESULTS

### Immortalized MSC show enhanced proliferation while maintaining surface marker expression and immunomodulatory function

**Figure 2:**
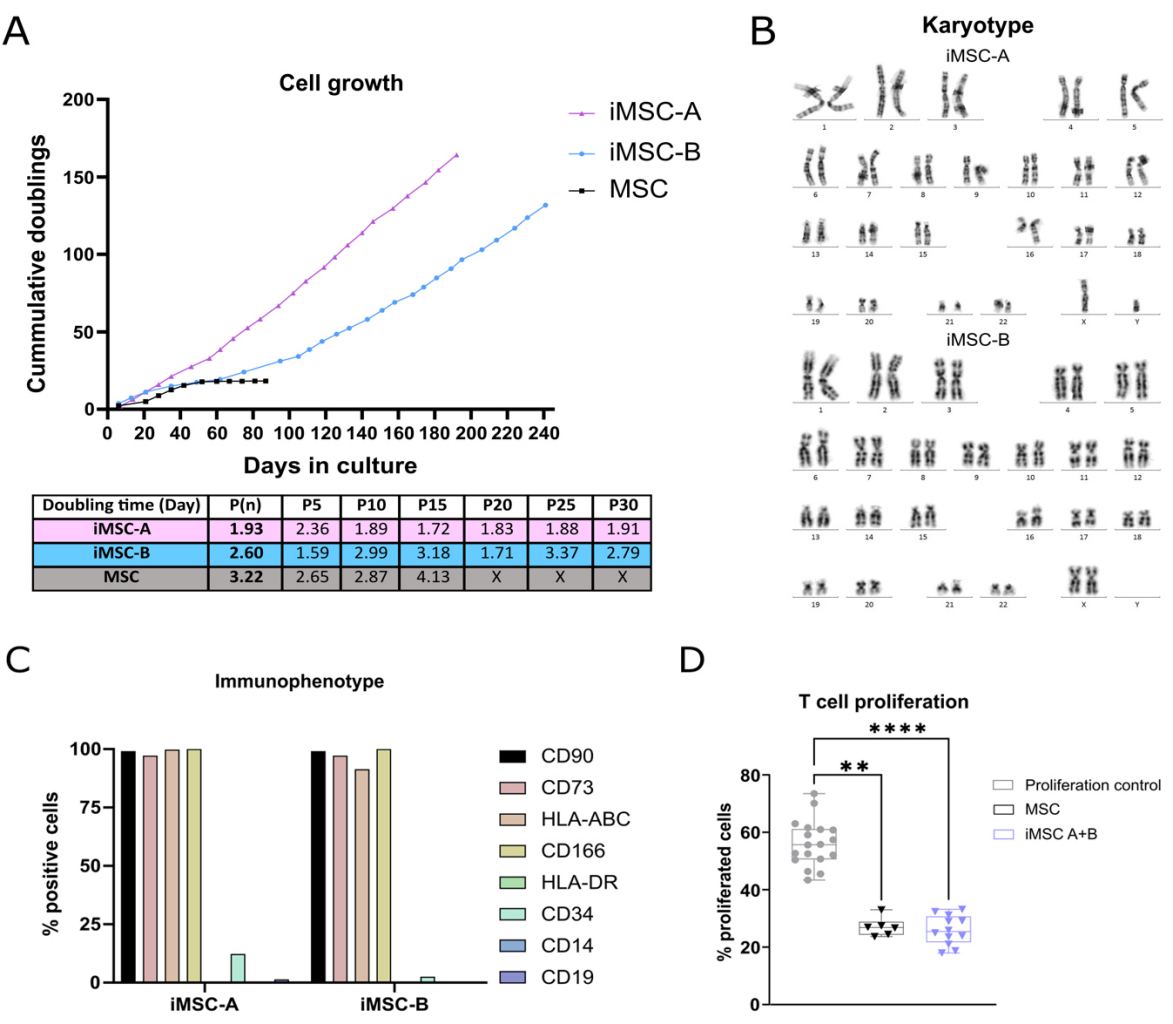
(A) Growth rate of the two immortalized MSC (iMSC) lines tested, expressed as cumulative doublings; (B) Karyotype of both iMSC lines; (C) Immunophenotype of the iMSCs of expected and unexpected markers of a MSC lineage analyzed by flow cytometry; (D) Inhibition of T cell proliferation by MSC and iMSC lines. Data from 3 independent experiments with a minimum of 2 replicates/condition. Data shows at least three independent experiments in each group. Comparison between groups was performed by one-way ANOVA followed by the Kruskal-Wallis test. Error bars represent SD. ** *P* < 0.01, **** *P* < 0.0001.

Established immortalized MSC lines (iMSC) from two different cell donors (named MSC-A and MSC-B in this manuscript) were used for this study to reduce batch-to-batch variability, improve yields, and reduce senescence during procedures. Indeed, iMSC lines maintained a steady proliferative rate (iMSC-A: 3 doublings/week, iMSC-B: 2 doublings/week) during the follow-up period (iMSC-A: 180 days, iMSC-B: 240 days), whereas primary MSCs showed a decline in the proliferative rate at 40 days of culture [Figure 2A]. Given the enhanced proliferation rates, we analyzed iMSC lines’ karyotypes to check for genomic aberrations in cell culture. iMSC-A line presented a partial chromosome 16q duplication (add(16)(q24)), while iMSC-B did not present any alteration in the chromosomal structure [Figure 2B], thereby confirming low alterations due to the immortalization step. In this line, MSC immortalization was performed by transfecting the hTERT gene (as explained in the “Cell culture and immortalization procedures” section). To discard the potential loading of the transcript onto EV, we checked its expression in cells and EVs by qPCR. The expression of hTERT in immortalized cell lines was significantly higher than in primary cells [Supplementary Figure 2] as assessed by RT-PCR. In the case of primary cell cultures, the crossing point (CP) was very close to the detection limit in our design (CP = 34.50). As for iMSC-EVs, they exhibit an even lower expression of hTERT compared to primary cell cultures (CP = 34.76 in iMSC-A-EVs, CP = 35 in iMSC-B-EVs). Transfer of hTERT to primary cell cultures was tested by culturing primary lung-derived fibroblasts for 6 h in the presence of iMSC-EVs. By qPCR of the hTERT mRNA, we did not find expression in primary fibroblast after incubation [Supplementary Figure 2B].

Immunophenotyping showed that both iMSC lines maintained primary MSC marker expression [Figure 2C], being positive for CD90, CD73, HLA-ABC, and CD166, and negative for HLA-DR, CD34, CD19, and CD14. Finally, to determine whether the immortalization procedure of MSC lines could affect their immunomodulatory capabilities, T cells were stimulated in the presence of primary MSC and iMSC lines, and their proliferation capacity was checked. The results confirmed that iMSC lines had comparable immunomodulatory capabilities on T cell proliferation as the primary MSC [Figure 2D], confirming no alteration in their immunomodulatory capacity due to immortalization.

### Hollow fiber cell bioreactor is suitable for iMSC culture

**Figure 3:**
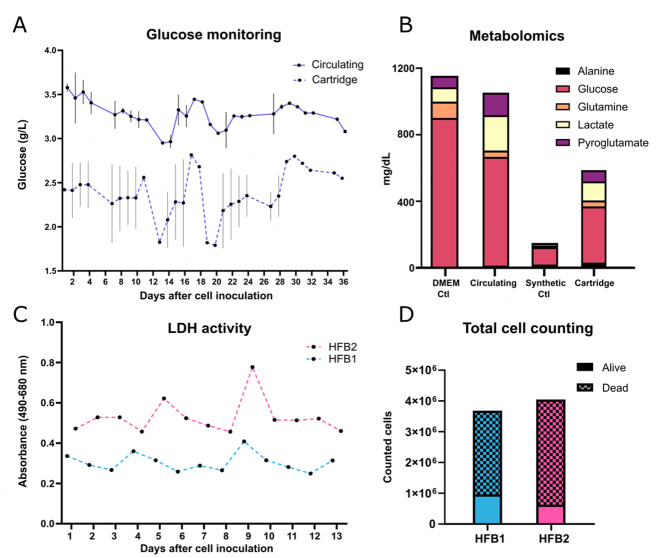
Cell growth monitoring. (A) Glucose monitoring of ECS medium (dashed line) and in the circulating medium (solid line). The data show the average of both bioreactors; (B) Metabolomics’ results showing different metabolite concentrations in culture medium control (DMEM Ctl), cartridge medium control (Synthetic Ctl), culture media after EV production (Circulating), and ECS medium after EV production (Cartridge). The data show the average of both bioreactors; (C) Lactate dehydrogenase (LDH) activity in the ECS of both hollow fiber bioreactors (HFB1 and HFB2); (D) Total count of detached cells, live and dead, from the two bioreactors. Error bars represent SD.

Given the maintenance of iMSC identity and function, we cultured both cell lines in hollow fiber bioreactors (HFB) for 4 weeks [Figure 1]. To monitor cell status during follow-up, we analyzed indirect biochemical markers as the seeding in the cartridge limits cell visualization. Glucose levels were measured daily inside the cartridge and in the circulating medium to monitor cell activity. Glucose levels decreased gradually during the first 15 days [Figure 3A], indicating active cell metabolism, and were constant afterwards in both compartments. Nutrient exchange between compartments is crucial for cell survival and could impact cell activity and the associated glucose consumption during follow-up. In this sense, glucose fluctuations inside the cartridge and in the circulating medium coincided, confirming a glucose exchange between both compartments. In addition, we performed metabolomic studies of culture media to further confirm nutrient exchange between compartments. Taking advantage of our design, we could compare the flow of some media components because we used a chemically defined medium on the cartridge and high-glucose DMEM on the circulating medium. The concentration of selected analyzed metabolites that were enriched in the circulating media DMEM increased in the harvested cartridge medium at the end of EV production compared to the control, corroborating the metabolite exchange between the compartments [Figure 3B].

Additionally, cellular cytotoxicity was studied by monitoring lactate dehydrogenase (LDH) levels inside the cartridge, which were stable throughout the follow-up, indicating no significant cell death [Figure 3C]. Finally, the total number of detached cells in supernatants at the end of EV production was calculated by combining daily counts from all supernatants harvested. The results revealed that most of the seeded cells from both HFB remained inside the cartridge, with a total count of 4 × 10^6^ (of the 3 × 10^7^ cells seeded) since the last day of EV production [Figure 3D].

Finally, immunophenotyping of recovered cells from the bioreactor showed that the iMSC lines maintained MSC marker expression after 30 days of culture inside the bioreactor [Supplementary Figure 3].

### Hollow fiber bioreactor iMSC-conditioned medium allows isolation of EVs

**Figure 4:**
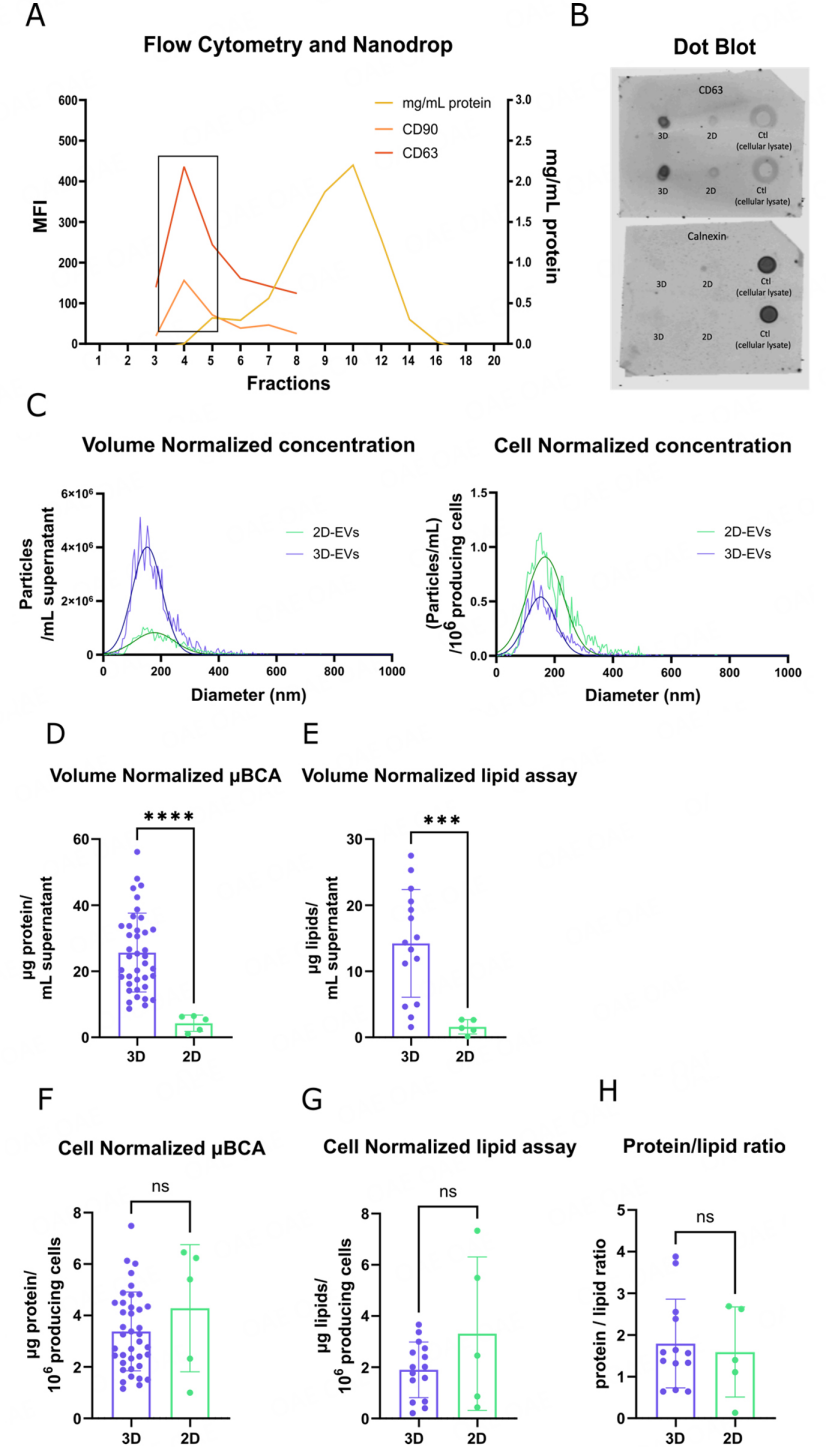
iMSC-EVs characterization. (A) Representative size exclusion chromatography (SEC) elution profile of iMSC-EV, positive for the EV marker CD63 and MSC marker CD90. Protein elution was measured at 280 nm absorbance by nanodrop and occurred later and separated from EV fractions; (B) Dot blot results for the detection of CD63 (top) and calnexin (bottom). 3D-derived samples from different weeks of EV-production of both bioreactors and 2D-derived samples from both iMSC lines were analyzed. The control (cellular lysate) is represented in duplicates; (C) Nanoparticle tracking analysis (NTA) showing the particle size distribution of 2D- and 3D-derived EVs. Data are normalized by mL of supernatant (left) or by million of producing cells (right); (D) iMSC-EV protein quantification by µBCA assay, showing the µg/mL of protein and (E) lipid quantification by Sulfo-phospho-vanillin (SPV) assay, showing the of µg/mL lipids, results normalized per mL of supernatant; (F) iMSC-EV protein quantification by µBCA assay, showing the µg/mL of protein and (G) lipid quantification by Sulfo-phospho-vanillin (SPV) assay, showing the of µg/mL lipids, results normalized per million producing cells. The samples represented are from two bioreactors (3D) and from five 2D-derived EV productions (2D); (H) Protein/lipid ratio for 3D and 2D samples. Bars represent means, and error bars represent SD. Comparison between groups was performed by Mann-Whitney Test. ns *P* > 0.05, *** *P* < 0.001, **** *P* < 0.0001.

As detailed in the methods section, iMSC-EVs were enriched by conditioned media ultrafiltration and size exclusion chromatography (SEC). SEC fractions were profiled by assessing the total protein content and expression of CD90 and CD63 to identify EV-enriched fractions. Expression peaks of both surface markers were found within fractions 3 to 6 [representative graph: Figure 4A], in concordance with previous results using 2D isolated EVs from primary culture^[24]^. The bulk of proteins were detected from fraction 10 onwards. In addition, dot blot assay discarded the presence of calnexin (as a reference for contaminating proteins) in EV fractions from different bioreactor productions, while confirming the presence of CD63 in all EV samples [Figure 4B]. Nanoparticle tracking analysis (NTA) was used to quantify particle concentration in HFB and 2D derived samples and assess particle size and zeta potential [Figure 4C]. HFB showed an increase in particle concentration per mL of CM compared to 2D samples (8.75 × 10^10^ and 2.07 × 10^10^ particles/mL, respectively). HFB samples compared to 2D-derived showed maintenance of EV size (150 and 175 nm, respectively) and zeta potential (-36.23 and -42.99 mV, respectively).

The specific protein content of the EV pools was assessed using µBCA assay. The results revealed that bioreactor-derived pools had approximately a 5-fold increase in protein (25.66 ± 11.93 *vs.* 4.283 ± 2.471 µg/mL) and lipid (13.64 ± 8.197 *vs.* 1.59 ± 1.082 µg/mL) content per mL of CM compared to 2D-derived pools [Figure 4D and E]. Higher EV yield, as measured by protein quantification and NTA, in HFB-derived EVs allows for easier scalability and downstream processing of samples. EV production per MSC was comparable both in protein and lipids [Figure 4F and G]., and particle concentration [Figure 4C] in HFB and 2D-derived samples. Notably, the EV pools from the HFB are purified from 24h conditioned medium (CM) productions, while the 2D pools are purified from 48h CM productions. Nevertheless, the protein/lipid ratio of each sample was comparable [Figure 4H], which, accordingly, with the maintained particle size and zeta potential, indicates a similar composition of both 2D and 3D-produced EVs. The HFB system showed high stability throughout the culture. While we observed a trend of reduced protein and lipid concentrations through the weeks, no significant differences were found in protein and lipids concentrations during the 4-week follow-up. In addition, protein/lipid ratios were stable during follow-up [Supplementary Figure 4]. Overall, this highlights an efficient process for the manufacturing of EVs, demonstrating a higher concentration of EVs in the bioreactor compared to the 2D cell culture, while maintaining 2D-EV characteristics.

### iMSC-EVs from bioreactors are phenotypically comparable to the 2D

**Figure 5:**
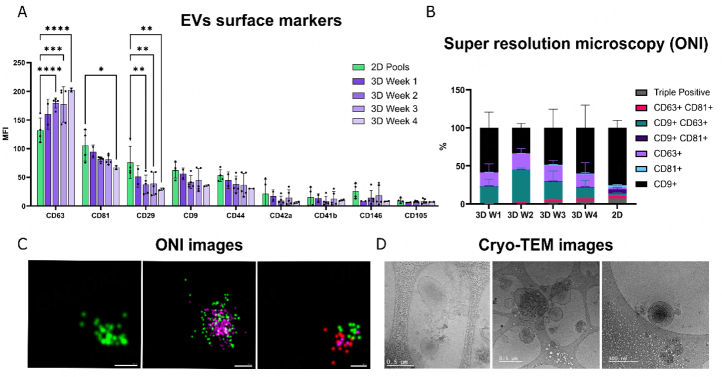
iMSC-EVs characterization. (A) MFI values of top expressed markers (CD63, CD81, CD29, CD9, CD44, CD146, CD42a, CD41b, CD105) from 2D- (pool) and 3D-derived samples determined by MACSPlex; (B) Super-resolution microscopy (ONI) results showing the percentage of particles expressing each tetraspanin (CD9, CD63, or CD81) and their co-expression in 2D and 3D samples; (C) ONI images of individual particles showing single (left), double (center), and triple positive (right) expression of tetraspanins (CD63: green, CD9: pink, CD81: red). Images are shown with 50 and 100 nm scale bars; (D) Cryo-TEM images of EV pools from different iMSC-EV bioreactor extractions. Images are shown with 0.5 μm scale bars. ns *P* > 0.05, * *P* < 0.05, ** *P* < 0.01， *** *P* < 0.001, **** *P* < 0.0001.

Next, bioreactor-derived EVs were characterized to investigate the maintenance of classical MSC markers compared to 2D-derived EV samples. Immunophenotyping EVs by bead-based flow cytometry showed that tetraspanins CD63, CD81, and CD9 were highly expressed in all samples. However, in contrast to the other most expressed markers, CD63 expression in EVs increased over the weeks of bioreactor cell culture. In addition, two characteristic MSC markers, CD29 and CD44, also showed high expression in both 3D- and 2D- produced EVs [Figure 5A]. CD29 expression was higher for 2D-derived EVs, while no significant differences were observed between the expression of bioreactor and 2D-produced EVs in CD44 and other markers (e.g., CD146, CD42a, CD41b, CD105).

We further analyzed EV at the single-particle level using super-resolution microscopy by labeling tetraspanins CD9, CD63, and CD81 [Figure 5B and C]. Results showed that the most expressed tetraspanin was CD9, especially in the 2D samples, being predominant above the other tetraspanins. In bioreactor-derived samples, we saw an increased expression of CD63 alone or in co-expression with CD9 [Figure 5B].

Finally, purified EVs’ morphology was examined using cryo-TEM. Microscopy pictures revealed the presence of high number of vesicles ranging from 80-500 nm [Figure 5D] in all samples.

### Bioreactor-derived iMSC-EV modulate angiogenesis by promoting HUVEC tube formation

To functionally characterize bioreactor-produced iMSC-EVs and test the capacity of iMSC-EVs to induce cell angiogenesis, matrigel-based tube-like formation assays were performed.

**Figure 6:**
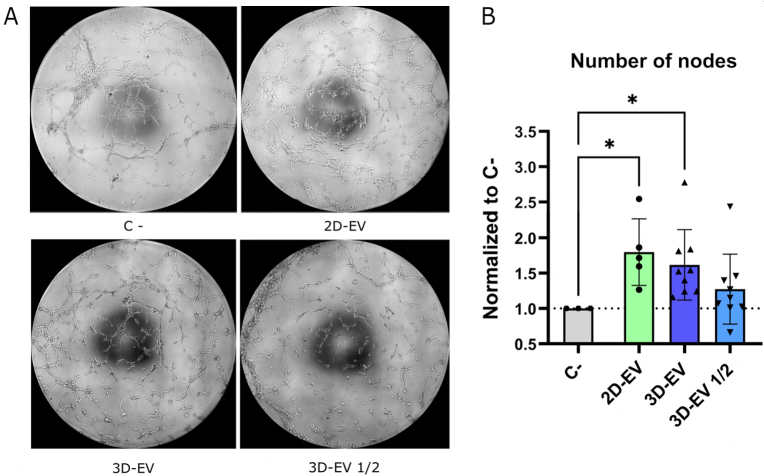
Matrigel-based tube-like formation assays. (A) Representative images (10x) of angiogenesis under each condition; (B) Quantification of angiogenesis as the number of nodes formed after 4 h of incubation with iMSC-EVs. Data are normalized by the negative control. Data from three independent experiments with 4 replicates/condition. Comparison between groups was performed by one-way ANOVA followed by the Kruskal-Wallis test. Error bars represent SD. ns *P* > 0.05, * *P* < 0.05.

iMSC-EVs derived from the hollow fiber bioreactor exhibited pro-angiogenic capabilities, promoting tube formation by human umbilical vein endothelial cells (HUVEC) in a dose-dependent manner [Figure 6A]. The number of nodes was higher compared to negative controls, while no significant differences were seen between the highest dose of 3D-EVs and 2D-derived EVs [Figure 6B].

## DISCUSSION

Mesenchymal stromal cell (MSC)-derived extracellular vesicles (MSC-EVs) hold great potential as advanced therapies for treating inflammation and boosting regeneration. Nevertheless, there is an unmet need to generate large, functional, and high-quality EV doses, to advance toward translation of EV-based cell-free therapies for preclinical and clinical application. In this study, we set up a novel workflow for the continuous production of large MSC-EV doses by culturing immortalized mesenchymal stromal cells (iMSCs) in a hollow fiber bioreactor (HFB), and tested their potential for the application *in vitro* and preclinical models with a focus on translation and scalability for clinical application.

Development of MSC biotherapeutics requires careful selection of cell sources along with the establishment of relevant cell culture conditions. Wharton’s jelly-derived MSCs (WJ-MSCs) have been thoughtfully studied and used for their higher proliferation rate and potential to modulate regeneration and immune modulation^[32,33]^. Nevertheless, replicative senescence is a common feature of primary cell culture, reducing yield and the possibility of long-term culture, thus requiring the availability of several cell donors, and introducing batch-to-batch variability in EV manufacturing. To overcome primary culture limitations, we generated two immortalized WJ-MSC lines by transfecting the human telomerase reverse transcriptase (hTERT).

MSC lines, or iMSCs, provided a stable, genetically, and phenotypically consistent cell population meeting the minimal criteria required for defining MSC by the International Society for Cellular Therapy (ISCT)^[34]^. Furthermore, iMSC functional potential was not altered, as demonstrated by a comparable suppressive immunomodulatory effect over T cell proliferation *in vitro* compared to the same donor primary MSC. These results are in line with other studies that have deeply characterized their immortalized MSC lines^[35-37]^.

While the immortalization approach addresses the imperative need to obtain clinically relevant cell numbers with minimal passages and doublings^[38]^, another concern associated with the use of iMSC lines was the potential transfer of the hTERT vector used for immortalization to EVs. In this work, we isolated EVs using size exclusion chromatography (SEC) which allows for higher purity grades with lower coisolation of mRNA transcripts compared to other approaches^[39,40]^. RT-qPCR experiments indicated minimal expression of hTERT in our SEC-isolated iMSC-EVs compared to the high expression of whole iMSC themselves. Moreover, the low expression levels in iMSC-EVs closely resembled those observed in primary MSC cells, diminishing concerns over safety. Other studies, contrarily to our results, reported hTERT mRNA to be highly secreted in EVs obtained using other isolation methods, such as ultracentrifugation of cultured human amniocytes conditioned media^[41]^, and Jurkat cell lines^[42]^, as well as precipitation-isolated tumor cells-derived EVs^[43,44]^. Furthermore, the same studies showed a functional effect on target cells mediated by the transfer of EV-associated hTERT mRNA.

Another matter of concern for the large-scale production of EVs is the application of scalable, GMP-adaptable platforms for cell culture. Hollow fiber bioreactors (HFBs) offer several advantages for EV production, including prolonged cell culture without additional passages, reduced batch-to-batch variability, and a more native-like microenvironment that supports cell functionality and interaction^[45]^. Moreover, the tissue-like cell culture conditions favor the formation of cell spheroids and may also help reduce culture costs and handling time compared to conventional 2D cell culture, and could increase EV yield^[46]^. In fact, we can extrapolate from previous reports that the maintenance of 30 million iMSC in a hollow fiber bioreactor requires 5 times less culture medium than when cultured in a CellSTACK-based system^[19]^.

To our knowledge, our study is the first in which iMSC lines have been used for large-scale EV production in a hollow fiber bioreactor. Compared to other works using HFB, we used different cell culture media in the extracapillary space (ECS) and the circulating media, which contained chemically defined media and standard serum-complemented media, respectively. This strategy reduces the cost of implementing chemically defined media in the whole system by combining the improved reproducibility and translation potential of synthetic serum-free media with the reduced costs and extensive literature of serum-supplemented media. To avoid contamination from serum-derived extracellular vesicles, cartridges used in this work had 20 kDa molecular weight cut-off pores that limit the filtration of extracellular particles, including EVs, toward the isolated supernatants. In this line, ultrafiltration of FBS has been recently described as an efficient way to deplete extracellular vesicles from FBS, with higher efficiency than ultracentrifugation and other commercial methods^[47]^. Moreover, we have not been able to quantify CD63+ particles in the empty cartridge with synthetic when we have circulating medium + FBS (data not shown). The use of serum-supplemented media in circulating media reduces costs to perform proof-of-concept experiments and is applicable to *in vitro* and preclinical models, and also allows for the study of metabolite flow between compartments. In this line, we could confirm by metabolomic study the homeostasis of metabolites between compartments, validating the use of the system for “long-term” iMSC culture with no disruption of metabolite balance and nutrient flow during continuous culture. Serum-derived molecules below the molecular weight cut-off will be able to flow to the isolated supernatants and their complete removal from the EV samples remains a challenge in the field. In the same line, the use of antibiotics should be avoided or minimized in the circulating medium, considering potential risks to patient safety and the development of antibiotic resistance. Hence, future studies are needed to comprehensively investigate the filtration of serum-derived molecules.

In comparison to other large-scale production systems, the HFB has a limitation regarding the capacity for direct visualization of the cells during the production period and the possibility to study their state. Nonetheless, our monitoring strategy based on glucose consumption and lactate dehydrogenase (LDH) release demonstrated consistent homeostasis throughout the incubation period. Glucose consumption was correlated in both the ECS and the circulating medium and showed a constant decrease during a minimum of two weeks of culture, while LDH levels did not significantly increase over the follow-up, indicating a balance between cell proliferation and death. Cell culture was stopped after 4 weeks of continuous culture due to no significant glucose consumption during the last week, which could be associated with diminished cell metabolism indicating an altered cell status. Nevertheless, although most cells remained trapped in the ECS, at the end of the follow-up, recovered iMSCs showed maintenance of classical MSC markers and high viability. The stability of the iMSC culture in the HFB system for 4 weeks is in line with previous studies involving primary MSC, where cells remained stable in the bioreactor for at least 30 days without changes in their phenotype or EV production capacity. However, other primary MSC studies showed different production times^[19,46,48,49]^, which could be associated with donor-to-donor variability and/or variable HFB culture settings.

One limitation of the HFB system to produce iMSC-EVs is the large cell number required for inoculation into the bioreactor, which must be expanded prior to production. However, to our knowledge, this is not unique to the HFB system, other large-scale productions systems similarly require extensive cell expansion before inoculation^[21,22,38]^

The small volume in which cells are cultured in the ECS of HFB allowed the large-scale production of EVs by SEC, which is not possible in other large-scale production systems, where cells are cultured in higher amounts of media. The optimized cell culture volume facilitated the pre-concentration step of the conditioned medium (CM) by ultrafiltration, reducing the downstream processing efforts.

To further analyze the limitations of the different large-scale production systems, comparative studies should be performed using the same conditions, including cell type, cell number, medium composition, surface coatings, among other factors. Key limitations that should be comparatively assessed in future studies, particularly for bioreactor scale-up, include the challenges of maintaining uniform cell growth and consistent nutrient distribution to prevent the formation of heterogeneous cell populations.

Once isolated, the composition, properties, and functionality of the iMSC-EVs produced in the bioreactor were compared using MISEV guidelines^[50]^ to the ones produced in 2D culture systems. Proper isolation of EVs from supernatants was confirmed by a combination of cryo-electron microscopy and surface marker phenotyping to visualize and characterize the EVs. Furthermore, our results showed no significant differences between the protein/lipid ratio from 3D and 2D culture-derived EVs, which demonstrated a comparable macromolecular composition and maintenance of the sample properties, and a balanced EV/contaminant content between conditions. In this line, protein and lipids were stable throughout the bioreactor follow-up, highlighting the reproducibility of the system. EV production in the HFB system showed an increased yield per volume of CM in terms of protein and particle count, which supports the scalability of the system, due to a reduced volume to be processed downstream to obtain high EV doses in a potential context of large-scale manufacturing. On the other hand, the particle count normalized per number of producing MSC was not significantly different between 2D and 3D (bioreactor) cultures. However, bioreactor CM isolation is performed every 24 h compared to 48 h in 2D-derived samples, which, if normalized by the time of production, would yield higher concentrations of protein (6.76 ± 3.06 *vs.* 4.28 ± 2.47 µg/mL per 1 × 10^6^ iMSC in 3D *vs.* 2D samples, respectively) and particles (2.36 × 10^10^ *vs.* 1.75 × 10^10^ particles/mL per 1 × 10^6^ iMSC in 3D *vs.* 2D samples, respectively) per iMSC in 3D samples in the same timeframe.

Moreover, we consider the strength of this 3D system to not rely on a single EV production. The hollow fiber bioreactor fills the gap of lacking cost-effective and scalable systems to produce MSC-EV for application in preclinical and clinical settings. It enables a high-throughput continuous production with minimal handling and reduces the volume that needs to be processed downstream.

Throughout the bioreactor production, we observed a slight decrease in the relative expression of the tetraspanins CD9 and CD81, as well as of the mesenchymal markers CD29 and CD44, over the weeks. On the contrary, CD63 expression slowly increased, showing its highest expression in the last week of production. Notably, we observed a low expression of immunogenicity markers such as HLA-DR and HLA-ABC antigens, also observed in other studies using hollow fiber bioreactors as MSC culture systems^[19]^, which further supports the notion that EVs produced by iMSC would exhibit low immunogenicity. Our results suggest a potential optimal window for EV production in hollow fiber bioreactor between the first and third week, where glucose consumption remains constant, to ensure product consistency.

To validate the functional properties of iMSC-EVs from HFB, we performed endothelial cell tube formation assays using human umbilical vein endothelial cells (HUVEC). EVs derived from different MSC sources cultured in 2D systems have been widely shown to induce angiogenesis *in vitro* and *in vivo* on different types of endothelial cells^[25,51-54]^. However, little is known about the maintenance of this functionality when MSC-EVs are produced in 3D culture systems. In this study, we demonstrated that iMSC-EVs from weeks 2 and 3 of culture produced in a HFB were able to induce HUVEC differentiation to tube-like structures *in vitro*, in a dose-dependent manner, and with a comparable functionality to same-line 2D cell culture-derived EVs. These observations are in line with EVs produced by MSC organoids, which have also been shown to induce angiogenesis, indicating no functional alteration due to the 3D culture system^[55]^.

While our study showed the potential and feasibility of implementing HFB systems involving immortalized cell lines and the combination of chemically defined media and serum-supplemented media to ensure reproducibility and reduce costs, there are some limitations for the direct application in GMP-grade manufacturing, namely the composition of circulating medium and potential molecule transfer to the ECS, and the potential transfer of hTERT via EVs, which was not significant in our syudy and show no transference to primary cells [Supplementary Figure 2].

In summary, these results collectively emphasize the potential of the hollow fiber bioreactor system in facilitating large-scale, scalable, GMP adaptable, functional, and high-quality EV production for clinical applications, showcasing its efficiency in terms of yield, time, and material costs. The extensive characterization presented in this study positions this production methodology as a candidate for standardizing EV production in the pursuit of preclinical and clinical applications.

## DECLARATIONS

### Copyright

© The Author(s) 2024.

## References

[1] Uccelli A, Moretta L, Pistoia V (2008). Mesenchymal stem cells in health and disease. Nat Rev Immunol.

[2] Le Blanc K, Frassoni F, Ball L, Developmental Committee of the European Group for Blood and Marrow Transplantation (2008). Mesenchymal stem cells for treatment of steroid-resistant, severe, acute graft-versus-host disease: a phase II study. Lancet.

[3] Duijvestein M, Vos AC, Roelofs H (2010). Autologous bone marrow-derived mesenchymal stromal cell treatment for refractory luminal Crohn's disease: results of a phase I study. Gut.

[4] Connick P, Kolappan M, Crawley C (2012). Autologous mesenchymal stem cells for the treatment of secondary progressive multiple sclerosis: an open-label phase 2a proof-of-concept study. Lancet Neurol.

[5] Cui LL, Kerkelä E, Bakreen A (2015). The cerebral embolism evoked by intra-arterial delivery of allogeneic bone marrow mesenchymal stem cells in rats is related to cell dose and infusion velocity. Stem Cell Res Ther.

[6] Volarevic V, Markovic BS, Gazdic M (2018). Ethical and safety issues of stem cell-based therapy. Int J Med Sci.

[7] Poltavtseva RA, Poltavtsev AV, Lutsenko GV, Svirshchevskaya EV (2019). Myths, reality and future of mesenchymal stem cell therapy. Cell Tissue Res.

[8] Wiklander OPB, Brennan MÁ, Lötvall J, Breakefield XO, El Andaloussi S (2019). Advances in therapeutic applications of extracellular vesicles. Sci Transl Med.

[9] van Niel G, D'Angelo G, Raposo G (2018). Shedding light on the cell biology of extracellular vesicles. Nat Rev Mol Cell Biol.

[10] García-Bernal D, García-Arranz M, Yáñez RM (2021). The current status of mesenchymal stromal cells: controversies, unresolved issues and some promising solutions to improve their therapeutic efficacy. Front Cell Dev Biol.

[11] Gimona M, Brizzi MF, Choo ABH (2021). Critical considerations for the development of potency tests for therapeutic applications of mesenchymal stromal cell-derived small extracellular vesicles. Cytotherapy.

[12] Patel DB, Gray KM, Santharam Y, Lamichhane TN, Stroka KM, Jay SM (2017). Impact of cell culture parameters on production and vascularization bioactivity of mesenchymal stem cell-derived extracellular vesicles. Bioeng Transl Med.

[13] Jiang Y, Jahagirdar BN, Reinhardt RL (2002). Pluripotency of mesenchymal stem cells derived from adult marrow. Nature.

[14] Li XY, Ding J, Zheng ZH, Li XY, Wu ZB, Zhu P (2012). Long-term culture in vitro impairs the immunosuppressive activity of mesenchymal stem cells on T cells. Mol Med Rep.

[15] Lian J, Lv S, Liu C (2016). Effects of serial passage on the characteristics and cardiac and neural differentiation of human umbilical cord Wharton’s jelly-derived mesenchymal stem cells. Stem Cells Int.

[16] Okamoto T, Aoyama T, Nakayama T (2002). Clonal heterogeneity in differentiation potential of immortalized human mesenchymal stem cells. Biochem Biophys Res Commun.

[17] Simonsen JL, Rosada C, Serakinci N (2002). Telomerase expression extends the proliferative life-span and maintains the osteogenic potential of human bone marrow stromal cells. Nat Biotechnol.

[18] Balducci L, Blasi A, Saldarelli M (2014). Immortalization of human adipose-derived stromal cells: production of cell lines with high growth rate, mesenchymal marker expression and capability to secrete high levels of angiogenic factors. Stem Cell Res Ther.

[19] Gobin J, Muradia G, Mehic J (2021). Hollow-fiber bioreactor production of extracellular vesicles from human bone marrow mesenchymal stromal cells yields nanovesicles that mirrors the immuno-modulatory antigenic signature of the producer cell. Stem Cell Res Ther.

[20] Zhang Y, Chopp M, Zhang ZG (2017). Systemic administration of cell-free exosomes generated by human bone marrow derived mesenchymal stem cells cultured under 2D and 3D conditions improves functional recovery in rats after traumatic brain injury. Neurochem Int.

[21] Haraszti RA, Miller R, Stoppato M (2018). Exosomes produced from 3D cultures of MSCs by tangential flow filtration show higher yield and improved activity. Mol Ther.

[22] Yan IK, Shukla N, Borrelli DA, Patel T

[23] Watson DC, Bayik D, Srivatsan A (2016). Efficient production and enhanced tumor delivery of engineered extracellular vesicles. Biomaterials.

[24] Monguió-Tortajada M, Roura S, Gálvez-Montón C (2017). Nanosized UCMSC-derived extracellular vesicles but not conditioned medium exclusively inhibit the inflammatory response of stimulated T cells: implications for nanomedicine. Theranostics.

[25] Clos-Sansalvador M, Garcia SG, Morón-Font M (2022). N-Glycans in immortalized mesenchymal stromal cell-derived extracellular vesicles are critical for EV-cell interaction and functional activation of endothelial cells. Int J Mol Sci.

[26] https://karger.com/books/book/358/ISCN-2020An-International-System-for-Human.

[27] https://www.fibercellsystems.com/.

[28] Monguió-Tortajada M, Morón-Font M, Gámez-Valero A, Carreras-Planella L, Borràs FE, Franquesa M (2019). Extracellular-vesicle isolation from different biological fluids by size-exclusion chromatography. Curr Protoc Stem Cell Biol.

[29] Visnovitz T, Osteikoetxea X, Sódar BW (2019). An improved 96 well plate format lipid quantification assay for standardisation of experiments with extracellular vesicles. J Extracell Vesicles.

[30] Verta R, Saccu G, Tanzi A (2023). Phenotypic and functional characterization of aqueous humor derived extracellular vesicles. Exp Eye Res.

[31] Skovronova R, Grange C, Dimuccio V, Deregibus MC, Camussi G, Bussolati B (2021). Surface marker expression in small and medium/large mesenchymal stromal cell-derived extracellular vesicles in naive or apoptotic condition using orthogonal techniques. Cells.

[32] Kern S, Eichler H, Stoeve J, Klüter H, Bieback K (2006). Comparative analysis of mesenchymal stem cells from bone marrow, umbilical cord blood, or adipose tissue. Stem Cells.

[33] Marino L, Castaldi MA, Rosamilio R (2019). Mesenchymal stem cells from the Wharton's jelly of the human umbilical cord: biological properties and therapeutic potential. Int J Stem Cells.

[34] Dominici M, Le Blanc K, Mueller I (2006). Minimal criteria for defining multipotent mesenchymal stromal cells. the international society for cellular therapy position statement. Cytotherapy.

[35] Burk J, Holland H, Lauermann AF (2019). Generation and characterization of a functional human adipose-derived multipotent mesenchymal stromal cell line. Biotechnol Bioeng.

[36] Cao H, Chu Y, Zhu H (2011). Characterization of immortalized mesenchymal stem cells derived from foetal porcine pancreas. Cell Prolif.

[37] Siska EK, Weisman I, Romano J (2017). Generation of an immortalized mesenchymal stem cell line producing a secreted biosensor protein for glucose monitoring. PLoS One.

[38] Hanley PJ, Mei Z, Durett AG (2014). Efficient manufacturing of therapeutic mesenchymal stromal cells with the use of the Quantum Cell Expansion System. Cytotherapy.

[39] Gámez-Valero A, Monguió-Tortajada M, Carreras-Planella L, Franquesa Ml, Beyer K, Borràs FE (2016). Size-exclusion chromatography-based isolation minimally alters extracellular vesicles' characteristics compared to precipitating agents. Sci Rep.

[40] Benedikter BJ, Bouwman FG, Vajen T (2017). Ultrafiltration combined with size exclusion chromatography efficiently isolates extracellular vesicles from cell culture media for compositional and functional studies. Sci Rep.

[41] Radeghieri A, Savio G, Zendrini A (2017). Cultured human amniocytes express hTERT, which is distributed between nucleus and cytoplasm and is secreted in extracellular vesicles. Biochem Biophys Res Commun.

[42] Likonen D, Pinchasi M, Beery E (2022). Exosomal telomerase transcripts reprogram the microRNA transcriptome profile of fibroblasts and partially contribute to CAF formation. Sci Rep.

[43] Goldvaser H, Gutkin A, Beery E (2017). Characterisation of blood-derived exosomal hTERT mRNA secretion in cancer patients: a potential pan-cancer marker. Br J Cancer.

[44] Gutkin A, Uziel O, Beery E (2016). Tumor cells derived exosomes contain hTERT mRNA and transform nonmalignant fibroblasts into telomerase positive cells. Oncotarget.

[45] Ahmed S, Chauhan VM, Ghaemmaghami AM, Aylott JW (2019). New generation of bioreactors that advance extracellular matrix modelling and tissue engineering. Biotechnol Lett.

[46] Tasma Z, Hou W, Damani T https://www.researchsquare.com/article/rs-1362793/v1.

[47] Kornilov R, Puhka M, Mannerström B (2018). Efficient ultrafiltration-based protocol to deplete extracellular vesicles from fetal bovine serum. J Extracell Vesicles.

[48] Jakl V, Ehmele M, Winkelmann M (2023). A novel approach for large-scale manufacturing of small extracellular vesicles from bone marrow-derived mesenchymal stromal cells using a hollow fiber bioreactor. Front Bioeng Biotechnol.

[49] Cao J, Wang B, Tang T (2020). Three-dimensional culture of MSCs produces exosomes with improved yield and enhanced therapeutic efficacy for cisplatin-induced acute kidney injury. Stem Cell Res Ther.

[50] Théry C, Witwer KW, Aikawa E (2018). Minimal information for studies of extracellular vesicles 2018 (MISEV2018): a position statement of the International Society for Extracellular Vesicles and update of the MISEV2014 guidelines. J Extracell Vesicles.

[51] Monguió-Tortajada M, Prat-Vidal C, Moron-Font M (2021). Local administration of porcine immunomodulatory, chemotactic and angiogenic extracellular vesicles using engineered cardiac scaffolds for myocardial infarction. Bioact Mater.

[52] Sharma M, Bellio MA, Benny M (2022). Mesenchymal stem cell-derived extracellular vesicles prevent experimental bronchopulmonary dysplasia complicated by pulmonary hypertension. Stem Cells Transl Med.

[53] Lu G, Su X, Wang L (2023). Neuroprotective effects of human-induced pluripotent stem cell-derived mesenchymal stem cell extracellular vesicles in ischemic stroke models. Biomedicines.

[54] Gangadaran P, Rajendran RL, Lee HW (2017). Extracellular vesicles from mesenchymal stem cells activates VEGF receptors and accelerates recovery of hindlimb ischemia. J Control Release.

[55] Kim J, Kim EH, Lee H, Sung JH, Bang OY (2023). Clinical-scale mesenchymal stem cell-derived extracellular vesicle therapy for wound healing. Int J Mol Sci.

