# Supplementary material for: Hollow fiber bioreactor allows sustained production of immortalized mesenchymal stromal cell-derived extracellular vesicles
Source: Extracell Vesicles Circ Nucl Acids. 2024 May 14;5(2):201–20. doi: 10.20517/evcna.2023.76 (PMC11648467; doi:10.20517/evcna.2023.76)
Supplement: Supplementary file 1 [file evcna-5-2-201-SupplementaryMaterials.pdf]

Supplementary Materials

Hollow fiber bioreactor allows sustained production of immortalized mesenchymal stromal cell-derived extracellular vesicles

Sergio G Garcia[1,2,#], Marta Sanroque-Muñoz[1,3,#], Marta Clos-Sansalvador[1,2], Miriam Font-Morón[1], Marta Monguió-Tortajada[1], Francesc E. Borràs[1,4], Marcella Franquesa[1,*]

[1]REMAR-IGTP Group, Health Science research Institute Germans Trias i Pujol (IGTP), Can Ruti Campus, Badalona 08916, Spain.

[2]Department of Cell Biology, Physiology and Immunology, Universitat Autònoma de Barcelona (UAB), Bellaterra 08193, Spain.

[3]Department of Biochemistry and Cell Biology, Universitat Autònoma de Barcelona (UAB), Bellaterra 08193, Spain.

[4]Department of Cell Biology, Physiology and Immunology, Universitat de Barcelona (UB), Barcelona 08028, Spain.

[#]Authors contributed equally.

***Correspondence to:** Dr. Marcella Franquesa, REMAR-IGTP Group, Health Science research Institute Germans Trias i Pujol (IGTP), Campus Can Ruti, Carretera de Can Ruti, Camí deles Escoles s/n, Badalona 08916, Spain. E-mail: mfranquesa@igtp.cat

© The Author(s) 2020. Open Access This article is licensed under a Creative Commons Attribution 4.0 International License (https://creativecommons.org/licenses/by/4.0/), which permits unrestricted use, sharing, adaptation, distribution and reproduction in any medium or format, for any purpose, even commercially, as long as you give appropriate credit to the original author(s) and the source, provide a link to the Creative Commons license, and indicate if changes were made.

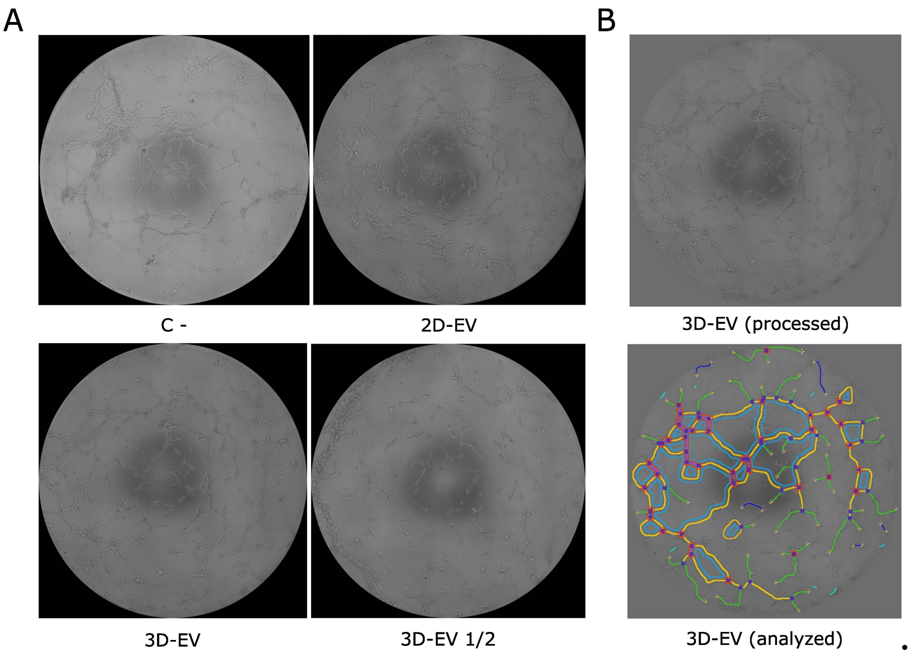

Supplementary Figure 1. Angiogenesis images analysis. (A) Original images of angiogenesis results. (B) Processed and analyzed images using imageJ program. Nodes are surrounded by a red line.

A

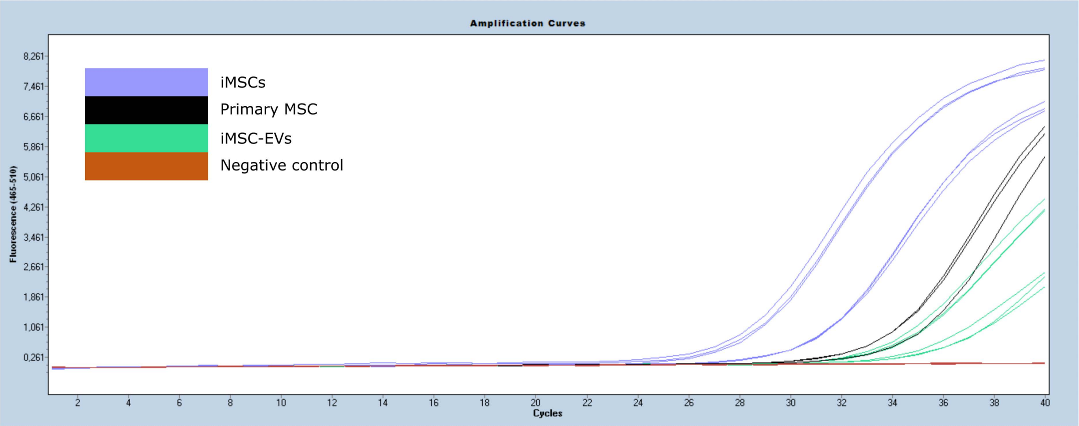

Sample	Number of initial cells	Total RNA (ng)	Used RNA for RT-PCR (ng)	Used RNA for qPCR (ng)	Equivalent cells for qPCR	CP
**Primary MSC**	9.00E+06	232.5	100	1.54	9935	34.5
**iMSC-A**	9.00E+06	104885	100	1.54	132	30.99
**iMSC-B**	9.00E+06	168640	100	1.54	82	28.3
**iMSC-A-EVs**	9.00E+06	89.5	38.5	1.54	154860	34.76
**iMSC-B-EVs**	1.50E+06	107.5	38.5	1.54	128930	35

B

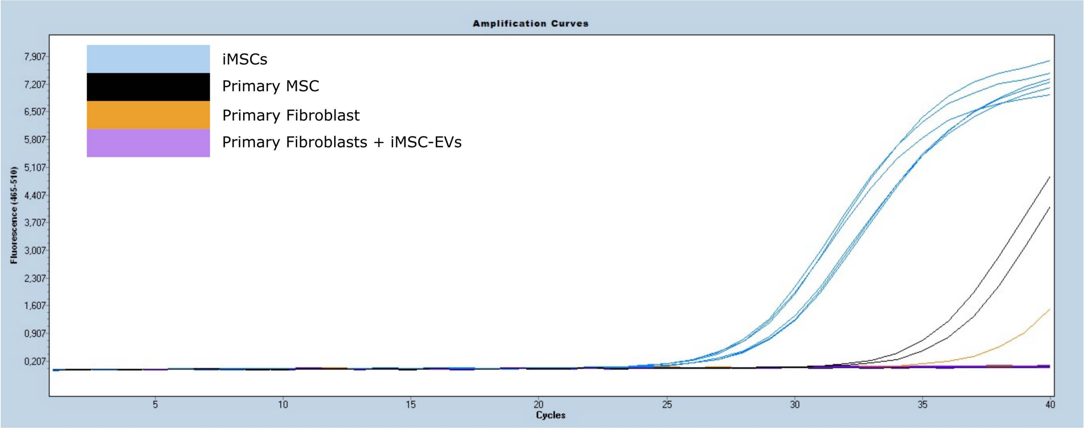

Sample	Number of initial cells	Total RNA (ng)	Used RNA for RT-PCR (ng)	Used RNA for qPCR (ng)	Equivalent cells for qPCR	CP
**Primary MSC**	1.50E+06	232.5	100	1.54	9935	34.5
**iMSC-A**	9.00E+06	104885	100	1.54	132	30.99
**iMSC-B**	9.00E+06	168640	100	1.54	82	28.3
**Primary fibroblasts**	1.00E+05	2596.8	100	2	77	>35
**Primary fibroblasts + iMSC-A-EVs**	1.00E+05	1103.4	100	2	181	>35
**Primary fibroblasts + iMSC-B-EVs**	1.00E+05	1013.1	100	2	197	>35

Supplementary Figure 2. RT-qPCR. (A) qPCR hTERT expression curves in primary MSC (black line), iMSC lines (blue lines), iMSC-EVs (green lines) and the negative control (red lines). The table shows the number of initial cells utilized for RNA extraction and the normalized CP for each sample. (B) qPCR hTERT expression curves in primary MSC (black line), iMSC lines (blue lines), primary fibroblasts (orange lines), primary fibroblasts incubated with iMSC-EVs (purple lines) and the negative

control (red lines). The table shows the number of initial cells utilized for RNA extraction and the normalized CP for each sample.

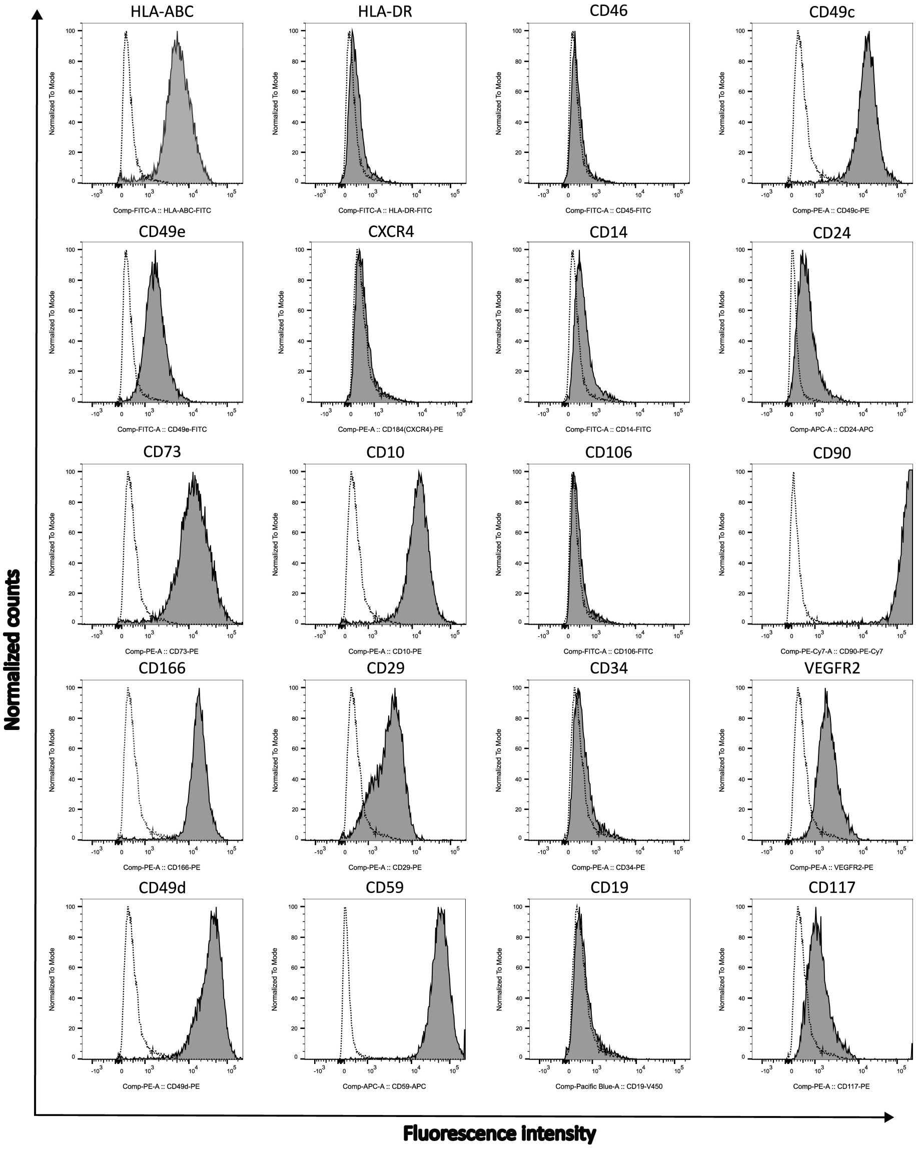

Supplementary figure 3. Immunophenotyping post bioreactor culture. Flow cytometry analysis of selected MSC surface markers of extracted cells recovered from the bioreactor shown as fluorescence intensity.

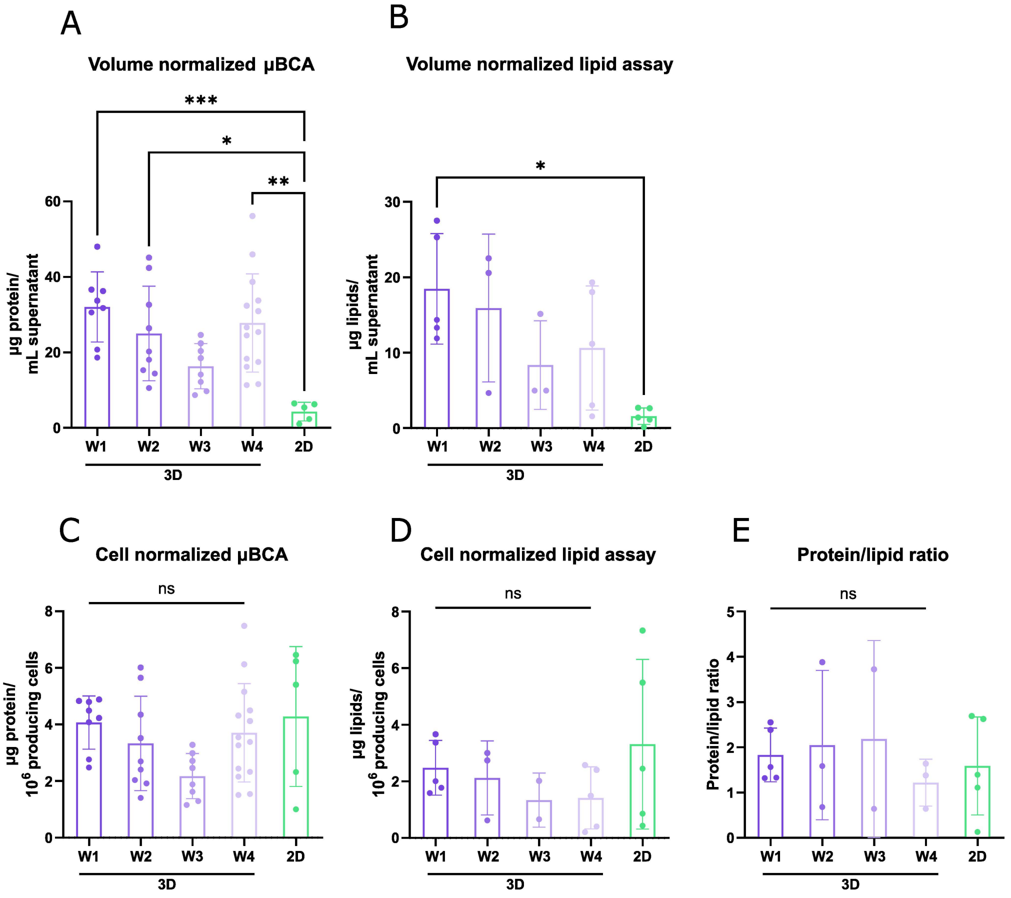

Supplementary Figure 4. iMSC-EVs characterization. (A) iMSC-EV protein quantification by μBCA assay, showing the μg/mL of protein and (B) lipid quantification by Sulfo-phospho-vanillin (SPV) assay, showing the of μg/mL lipids, results normalized per mL of supernatant. (C) iMSC-EV protein quantification by μBCA assay, showing the μg/mL of protein and (D) lipid quantification by Sulfo-phospho-vanillin (SPV) assay, showing the of μg/mL lipids, results normalized per million producing cells. The samples represented are from two bioreactors (3D) reported by weeks of EV production, and from five 2D-derived EV productions (2D). (E) Protein/lipid ratio for 3D and 2D samples. Bars represent means, error bars represent SD. Comparison between groups was performed by Mann-Whitney Test. ns $P > 0.05$, *** $P < 0.001$, **** $P < 0.0001$.
